# Comparing the Effects of FIFO/DIDO Workers Being Home versus Away on Sleep and Loneliness for Partners of Australian Mining Workers

**DOI:** 10.3390/clockssleep2010009

**Published:** 2020-03-06

**Authors:** Kerrie-ann I. Wilson, Sally A. Ferguson, Amanda Rebar, Kristie-Lee Alfrey, Grace E. Vincent

**Affiliations:** Central Queensland University, Appleton Institute of Behavioural Science, Adelaide 5034, South Australia, Australia; kerrieannwilson@gmail.com (K.-a.I.W.); sally.ferguson@cqu.edu.au (S.A.F.); a.rebar@cqu.edu.au (A.R.); k.alfrey@cqumail.com (K.-L.A.)

**Keywords:** co-sleeping, fly in fly out: drive in drive out FIFO/DIDO, loneliness, mining, sleep duration, sleepiness, sleep quality

## Abstract

Fly in Fly out/Drive in Drive out (FIFO/DIDO) is a prevalent work arrangement in the Australian mining industry and has been associated with adverse outcomes such as psychological stress, sleep disturbances, fatigue, and work/life interference. FIFO/DIDO work arrangements have the potential to not only impact the FIFO/DIDO worker, but also the partner of the FIFO/DIDO worker. However, there is sparse empirical evidence on the impact of FIFO/DIDO work arrangements on partners’ sleep and subsequent performance. Therefore, the primary aim of this study was to describe and compare partners’ sleep quality, sleep duration, sleepiness, and loneliness when the FIFO/DIDO workers were at home (off-shift) and away (on-shift). A secondary aim of this study was to examine whether differences in partners’ sleep quality and sleep duration as a result of FIFO/DIDO worker’s absence could be partially explained through the presence of dependents in the home, relationship duration, chronotype, duration in a FIFO/DIDO role, and loneliness. Self-reported questionnaires were completed by 195 female and 4 male participants, mostly aged between 18 and 44 years and who had been in a relationship with a FIFO/DIDO mining worker for more than five years. Of note, most participants subjectively reported poor sleep quality, insufficient sleep duration, excessive sleepiness, and moderate to extreme loneliness compared to the general population regardless of whether the FIFO/DIDO workers were at home or away. Compared to when the FIFO/DIDO workers were at home, partners experienced reduced sleep quality and increased loneliness when the FIFO/DIDO workers were away. Secondary analyses revealed that loneliness may partially underpin the negative effect that FIFO/DIDO workers’ absence has on sleep quality. Further research is needed to understand the factors that contribute to poor sleep quality, insufficient sleep duration, excessive sleepiness, and loneliness of FIFO/DIDO partners to inform appropriate strategies to support FIFO/DIDO partners’ health and wellbeing not only in the mining population, but other industries that incorporate similar FIFO/DIDO work arrangements (e.g., emergency services, offshore drilling, and transport).

## 1. Introduction

The mining industry is one of the largest and most dynamic industries in Australia, employing 1.8% (mostly male) of the Australian workforce [1]. Given that mining locations are often remote, daily commuting to work is impractical and has mostly been replaced with fly-in fly-out or drive-in drive-out (FIFO/DIDO) work arrangements [2]. FIFO/DIDO arrangements typically include a mix of 12 h day and night shifts where workers reside on-site in temporary accommodation for the duration of their work period (between 1–4 weeks) and then return to their usual place of residency during their off-shift period [3,4].

There are benefits for families in FIFO/DIDO working arrangements including high income and the ability to maintain an urban lifestyle with more facilities and services available without relocating the family to rural/remote locations [5]. Whilst the mining workforce has the highest median weekly earnings in the Australia working population, the roles come at a cost as they are often reported to be related to long, physically demanding shifts in isolated locations away from the workers’ homes [6]. FIFO/DIDO work arrangements can interfere with home and family life in a number of ways. The physical distance between work and home life can leave some workers feeling like they are displaced from family, friends, and social networks [7]. In addition, some FIFO/DIDO workers report difficulties forming and maintaining long-term relationships, finding time to get things done at home, and fulfilling family responsibilities [8]. Physical and psychological distance is a reported source of tension for FIFO/DIDO workers who report often missing family events and feeling a sense of disconnect with their partners [9]. Importantly, the impacts of FIFO/DIDO arrangements on work and family life have been reported not only by the worker, but also by the worker’s partner [9,10]. One key impact is disturbances to sleep [8].

Inadequate sleep (e.g., short sleep duration, poor sleep quality) is linked with adverse physical and psychological outcomes including cardiovascular disease, obesity, diabetes, memory impairment, and depression [11,12,13]. Until recently, the evidence relating to sleep disturbances for workers and partners in the FIFO/DIDO context was largely subjective [8]. A study examining health behaviours of FIFO workers and partners using self-report measures found that workers and partners reported poorer sleep quality, poorer nutrition, less relaxation time, reduced exercise, and smoked more cigarettes when the partner was away compared to when they were home [14]. A second study reported that partners experience similar levels of fatigue as the worker across the FIFO/DIDO roster and found partners’ fatigue and depression were worst during the middle of shift times when the worker was away [15]. 

Psychological distress in mining workers has been found to be higher than the average Australian day worker [7]. Further, both FIFO/DIDO workers and their partners have reported feelings of isolation, loneliness, and tiredness [9,16,17]. Loneliness has been linked to increased rates of cognitive decline, which is associated with increased blood pressure and can affect everyday life, including sleep [18,19]. Further, people who feel lonely tend to exhibit decreased sleep efficiency (i.e., less total sleep within the sleep period) and greater impairments in daytime functioning than individuals who experience a normal level of loneliness, even in instances where duration of sleep is the same [20]. For example, individuals with excessive daytime sleepiness experience difficulties with cognitive, emotional, and physical functioning. This degree of sleepiness negatively affects performance at home and work, increases the risk of accidents, and decreases social participation and quality of life [21]. There is no literature examining the impact of loneliness on sleep or daytime sleepiness in FIFO/DIDO workers or partners. Further, there has been no research on the sleep of FIFO/DIDO partners that considers other influences such as presence of dependents, relationship duration, chronotype (i.e., morningness-eveningness trait), and duration in the FIFO/DIDO role. 

The primary aim of this study was to explore partners’ sleep quality, sleep duration, sleepiness, and loneliness when the FIFO/DIDO workers were at home compared to away. The secondary aim of this study was to examine potential influences on partners’ sleep quality and sleep duration. It was hypothesised that partners would report greater sleep quality and duration and reduced sleepiness and loneliness when the FIFO/DIDO workers were at home compared to away. It is also hypothesised that any negative effects of FIFO/DIDO workers’ absence on partners’ sleep will be underpinned partially by having dependents in the home, relationship duration, chronotypes, duration in the FIFO/DIDO role, and loneliness. 

## 2. Method and Analysis 

### 2.1. Study Design and Recruitment Procedures

This was a cross-sectional study conducted using an online questionnaire (Survey Monkey©). Partners of mining industry workers were targeted for recruitment given that FIFO/DIDO work arrangements are prevalent throughout this sector. Participants were recruited through mining-related social media (e.g., Facebook) communities from July–August 2019. Participants completed informed consent confirming that they were over 18 years, currently residing in Australia, a current partner of a FIFO/DIDO worker in mining related industry, and had read and understood the study information sheet. Ethics approval was obtained from Central Queensland University Human Research Ethics Committee (2019-022).

### 2.2. Participants

Two hundred and thirteen participants commenced the survey; however, data from two people were excluded because the participants were neither in a current relationship nor cohabitating with a FIFO/DIDO mining industry worker. Twelve people only answered the basic demographic questions, which did not allow for sufficient data for imputation (process outlined in statistical analyses section), so their data were excluded. This resulted in a final sample size of 199 participants with 195 females (98%) and 4 males (2%). 

### 2.3. Measures

#### 2.3.1. Basic Demographics

Demographic characteristics included questions regarding: gender (male, female, other), age (years), number of household members (age categories), relationship duration (e.g., less than 1 year, 1–2 years, 2–3 years, 3–5 years, 5–10 years, and 10+ years), and employment status (e.g., full-time, part-time, self employed, casual, student, household duties). Number of household members was coded into two categories for dependents under the age of 18 in the home (no dependents, dependents). Relationship duration was coded into three categories: 0–2 years, 2–5 years, and 5 or more years. 

#### 2.3.2. FIFO/DIDO Work Arrangements

FIFO/DIDO characteristics included questions regarding how long the worker had been employed in a FIFO/DIDO role (years) and how many consecutive days the worker was away for work (e.g., < 3 days, 3 days, 4 days, 5 to 7 days, 8 to 14 days, and > 2 weeks). The duration in a FIFO/DIDO role was coded into three categories: 0–2 years, 2–5 years, and 5 or more years. 

#### 2.3.3. Sleep Arrangements

To understand co-sleeping arrangements, participants were asked questions including: does your partner sleep in the same bed when he/she is at home (always, usually, sometimes, rarely, and never), is your sleep disrupted by your partner’s sleep habits (always, usually, sometimes, rarely, and never), do you feel your sleep is any different when your partner is at home compared to when away (I get better sleep when partner is away, I get better sleep when partner is at home, or there is no difference to my sleep), and how long does it take for your sleep to return to normal when your partner leaves (number of nights) and when your partner returns (number of nights). Questions to inform whether bed partners had matching chronotypes included: are you a morning or evening type of person and which type do you consider your partner to be (i.e., definitely a morning type, rather more a morning type than an evening type, rather more an evening than a morning type, and definitely an evening type). These responses were categorised into matched or unmatched chronotype where the partner and worker were both morning or evening types. 

#### 2.3.4. Sleepiness

The Epworth Sleepiness Scale [22] is a simple and effective tool that provides a subjective measure of daytime sleepiness reported to have high validity and reliability [23,24,25]. Eight items differentiate between average sleep and significant issues with sleep by asking how likely it is that an individual will doze in a specific situation, which is rated from 1 to 3 on a Likert scale where 3 indicates a high chance of dozing. The scores for the 8 situations were added together to give each participant a global score of 0–24. A global score > 9 is reflective of excessive sleepiness [22]. Partners were asked to report daytime sleepiness separately for when FIFO/DIDO workers were (a) away at work and (b) at home. 

#### 2.3.5. Loneliness

The University of California, Los Angeles Loneliness Scale provides a subjective and global measure of the phenomenon of loneliness, as well as feelings of social isolation [26,27,28]. The revised UCLA Loneliness Scale in a reduced form is reported to have high reliability and validity [27,29]. The 10-item scale has been reported to have internal consistency and convergent validity similar to the 20 item scale, is shorter, and provides superior model fit [30]. Participants rated each item on a scale from 1 (never) to 4 (often) and were asked to respond to each item for both shift periods (i.e., rate feelings of loneliness for when the FIFO/DIDO workers were away and rate feelings of loneliness for when the FIFO/DIDO workers were at home). The scores for the 10 items were summed to give each participant a global score of 0–40. Global scores > 21 are reflective of experiencing moderate to extreme loneliness [31].

#### 2.3.6. Sleep Quality and Sleep Duration

The Pittsburgh Sleep Quality Index (PSQI) was used to measure sleep quality and sleep duration [32]. The PSQI is a subjective measure of duration, quality, and patterns of sleep and has reported high validity and reliability in numerous studies [24,33,34]. The scale measures 7 components of sleep over the last month including subjective sleep quality and sleep duration. “Good” sleep is differentiated from “poor” sleep using a Likert scale of 0 to 3, where 0 is reflective of good and 3 is reflective of poor. Subscores for the 7 components were tallied to yield a global score of 0–21. A global sum of ≥ 5 is indicative of poor sleep quality [32]. Sleep duration was extracted from the PSQI item “how many hours of actual sleep do you get at night?” Partners were asked separate PSQI questions for when FIFO/DIDO workers were away at work and for when FIFO/DIDO workers were at home, resulting in two sets of distinct sleep quality and sleep duration scores per participant.

### 2.4. Statistical Analyses

Missing data patterns were investigated and imputed with the mice function in R [35,36]. No anomalies were detected so all available data were used for imputation, which was applied using Fully Conditional Specification [37] in which each missing variable is imputed by a separate model of continuous, binary, unordered categorical, and ordered categorical data. Missingness of variables ranged from 10%–36%, with sleepiness and loneliness being the most missing and sleep duration being the least. Prior to analyses, the data were checked for outliers and violations of assumptions [38]. Outliers with modest sway (*n* = 3) were winsorized to 3 standard deviations above or below the mean [39] and outliers with extreme sway (*n* = 2) were omitted (> 5 SD +/− M).

To compare sleep quality, sleep duration, sleepiness, and loneliness for when the FIFO/DIDO workers were away and at home, separate repeated-measures ANCOVA were used with the fixed factor of the worker being home versus away. To examine the independent effects of sleep quality, covariates of age and sleep duration were included. To examine the independent effects of sleep duration, covariates of age and sleep quality were included. To examine the independent effects of sleepiness, covariates of sleep quality, sleep duration, and loneliness were included. To examine the independent effects of loneliness, covariates of daytime functioning, sleep quality, and sleep duration were included. 

Hypotheses were further tested using linear regression modelling. First, to test for differences in sleep quality when workers were home compared to when they were away, a model was tested with sleep quality regressed onto a dichotomous home versus away nominal variable as well as the covariate of sleep duration to ensure that the findings were interpretable as predicting the variability in sleep quality independent from that explained by sleep duration. Second, to test if there were differences in sleep duration between when partners were home compared to away, a model was tested with sleep duration regressed onto home versus away and sleep quality. If there were significant home versus away effects in either model, an a priori theory-based stepwise approach was used to test potential underlying mechanisms for the difference. The original model (step 1) was extended to include dependents (step 2), relationship duration (step 3), chronotype match of partners (step 4), duration of FIFO role employment (step 5), and loneliness (step 6). A variable was considered a potential mechanism of the underlying home versus away difference if the step in which it was added to the model resulted in a significant change in the adjusted R^2^ and a reduction in the significance of the home versus away effect. Statistical significance was set at *p* < 0.05.

## 3. Results

### 3.1. Descriptive Results

#### 3.1.1. Demographic Characteristics

Most participants (74.9%) were aged between 18 and 44 years. The majority of participants were employed (71.3%) in casual, part-time, full-time, or self-employed roles. A smaller number, 25.6% (*n* = 51), of participants were not employed and attending to home duties. Most (77.9%, *n* = 155) participants reported having at least one dependent younger than 18 years in the household and (80.4%, *n* = 160) had been in the relationship with their FIFO/DIDO worker partner for five or more years. 

#### 3.1.2. FIFO/DIDO Work Characteristics

FIFO/DIDO workers typically were away from home between 1–4 weeks (49.7% worked away from home between 8–14 days and 32.7% worked away for 14+ days). More than half of the sample (63.4%, *n* = 126) had five or more years of experience with FIFO/DIDO work arrangements. 

#### 3.1.3. Sleeping Arrangements

Only a small number (18.1%, *n* = 36) of participants reported that they felt no difference to their sleep when the FIFO/DIDO workers were away or at home. However, 44% (*n* = 88) of participants felt that they slept better when the FIFO/DIDO workers were at home and 37.7% (*n* = 75) felt they slept better when the FIFO/DIDO workers were away. Only 7.5% (*n* = 15) of participants reported that the FIFO/DIDO workers’ sleep did not affect their own sleep. A large proportion (76.9%, *n* = 153) of participants reported that they always slept in the same bed as the FIFO/DIDO worker. 

Almost half (42.7%, *n* = 85) of participants reported having a synchronised chronotype with the FIFO/DIDO worker (i.e., morningness and morningness or eveningness and eveningness). Most participants (66.3%, *n* = 132) do not use sleeping aids, although a small number of participants reported using them only when the FIFO/DIDO workers were away (15.6%, *n* = 31), whilst 4.5% (*n* = 9) used sleeping aids only when the FIFO/DIDO workers were at home. 

When the FIFO/DIDO worker was away, 20.1% (*n* = 40) participants reported that their sleep never returns to normal, 32.7% (*n* = 65) reported that their sleep returns to normal after the first night, and only 14.1% (*n* = 28) reported that their sleep does not change. Similar findings were recorded for when the FIFO/DIDO workers returned and were at home, with 14.1% (*n* = 28) of participants reporting that their sleep never returns to normal, 40.7% (*n* = 81) reporting that their sleep returns to normal after the first night, and 15.6% (*n* = 31) reporting that their sleep does not change. 

#### 3.1.4. Sleep Quality and Sleep Duration

As shown in Table 1, 82.9% (*n* = 165) participants were poor sleepers when the FIFO/DIDO workers were at home and 93% (*n* = 185) of participants were poor sleepers (PSQI >5) when the FIFO/DIDO workers were away. When the FIFO/DIDO workers were away, participants reported on average less sleep duration (M ± SD, 6.9 ± 1.6 h) than when the FIFO/DIDO workers were at home (7.2 ± 1.5 h).

#### 3.1.5. Sleepiness

Table 1 presents sleepiness scores, revealing that participants reported excessive daytime sleepiness when the FIFO/DIDO workers were away (53.3%, *n* = 106) and at home (55.8%, *n* = 111). Participants were scored as dangerously sleepy (>16) when FIFO/DIDO workers were away at work (36.2%, *n* = 72) and at home (35.7%, *n* = 71).

#### 3.1.6. Loneliness

As depicted in Table 1, loneliness scores indicated that 44.2% (*n* = 88) of participants experienced extreme loneliness and 39.2% (*n* = 78) participants experienced moderate loneliness when the FIFO/DIDO workers were away. When the FIFO/DIDO workers were at home, more participants (43.7%, *n* = 87) reported moderate loneliness and less participants (12.1%, *n* = 24) reported extreme loneliness. 

#### 3.1.7. Bivariate Correlations of Sleep Quality, Sleep Duration, and Loneliness 

Associations between all variables were examined using correlation testing independent of whether the FIFO/DIDO mining workers were away or at home. Sleep quality was significantly inversely associated with sleep duration (*r* = −0.29, *p* < 0.001), meaning that as sleep quality scores increased (i.e., poorer sleep quality), sleep duration increased. Sleep quality was positively associated with loneliness (*r* = 0.32, *p* < 0.001), meaning that as sleep quality scores increased (i.e., poorer sleep quality), loneliness increased. Sleep duration was also significantly inversely associated with loneliness (*r* = −0.20, *p* < 0.001), suggesting that loneliness increased as sleep duration decreased. 

### 3.2. Testing Potential Mechanisms of Differences

#### 3.2.1. Sleepiness

As shown in Table 2, partners experienced excessive sleepiness when the FIFO/DIDO workers were away (15.3 ± 4.6 ESS) and at home (15.0 ± 4.1 ESS) and no significant difference was found between FIFO/DIDO workers being away or at home *f*(1,389) = 0.003, *p* = 0.96.

#### 3.2.2. Loneliness 

As shown in Table 2, there was a significant medium-large difference in partners’ loneliness between when the FIFO/DIDO workers were away at work and when the FIFO/DIDO workers were at home, *f*(1,389) = 65.33, *p* < 0.001, η_ρ_^2^ = 0.14. In support of the hypothesis, FIFO/DIDO partners experienced 5.02 (95% CI = 3.80, 6.25) more units of self-reported loneliness when the FIFO/DIDO workers were away compared to when the FIFO/DIDO workers were at home. The effects of the covariates of sleep quality *f*(1,389) = 20.80, *p* < 0.001, η_ρ_^2^ = 0.05, and sleep duration *f*(1,389) = 4.58, *p* = 0.033, η_ρ_^2^ = 0.01 were statistically significant.

#### 3.2.3. Sleep Quality and Sleep Duration

Initial testing reported a significant small-medium difference in sleep quality between FIFO/DIDO workers home versus away, *f*(1,390) = 12.12, *p* = 0.001, η_ρ_^2^ = 0.03. In support of the hypothesis, FIFO/DIDO partners experienced 1.08 (95% CI = 0.47, 1.69) less units of self-reported sleep quality when the FIFO/DIDO workers were away compared to when the FIFO/DIDO workers were at home. There was a signficant difference in partners’ sleep quality when FIFO/DIDO workers were home versus away (β = 0.17, *p* < 0.05), even after accounting for sleep duration. 

The a priori determined stepwise analyses revealed that this difference was not explained by dependents, duration of the relationship, whether the partners and FIFO/DIDO workers have the same chronotype, or the duration of time in the FIFO/DIDO role (Change in Adj. R^2^ = 0.01; F[1,387] = 3.45, *p* = 0.06). However, Step 6, which is shown in Table 3, revealed that loneliness may partially explain the difference in sleep quality between when FIFO/DIDO workers were home versus away. That is, loneliness statistically significantly predicted variability in sleep quality beyond the other predictors (Change in Adj. R^2^ = 0.05; F[1,386] = 23.30, *p* < 0.01), and the effect of home versus away was no longer statistically significant when loneliness was included in the model (β = 0.07, *p* = 0.18). 

Initial testing reported no significant difference in participant sleep duration between when FIFO/DIDO workers were away at work and at home (*p* = 0.486). There was no significant difference in sleep duration between when FIFO/DIDO workers were home versus away (β = −0.11, *p* = 0.49) after accounting for sleep quality; therefore, no follow-up stepwise analyses were conducted.

#### 3.2.4. Post-Hoc Exploratory Analyses

The hypotheses tested potential explanations for the effects of outcome differences when FIFO/DIDO workers were home versus away; however, an alternative option is that the differences were moderated (i.e., different depending on certain factors). For example, it could be that there were differences in sleep for home versus away periods only if couples had no dependents living in the house. As post-hoc exploratory analyses, it was tested whether moderation effects were present for workers’ home versus away differences in partners’ sleep quality or sleep duration. No significant moderation effects were found for dependents, duration of the relationship, whether the partner and worker have the same chronotype, the duration of time in the FIFO/DIDO role, or loneliness (all *p* < 0.05).

## 4. Discussion

The House of Representatives Standing Committee Regional Australia inquiry and the Child Family Community Australia review both found a lack of depth and breadth in current peer-reviewed literature regarding the impacts of FIFO/DIDO mining work arrangements on families and made recommendations for specific research to be undertaken [16,40]. Existing research suggests that detrimental effects to workers mental health, fatigue, and sleep can be found in similar travel-intense work arrangements such as emergency services, offshore drilling, and transport [41,42,43]. The House of Representatives Standing Committee Regional Australia specifically called for more research on how FIFO/DIDO work impacts health behaviours within the mining population to identify suitable strategies to minimise or cope with adverse health outcomes that can derive from the FIFO/DIDO lifestyle. 

In response to this call, the aim of this study was to explore the effects of FIFO/DIDO mining work arrangements on the sleep of FIFO/DIDO partners (i.e., sleep quality and sleep duration), sleepiness, and loneliness. The current study found no significant difference in FIFO/DIDO partners’ sleep duration when FIFO/DIDO workers were at home and when FIFO/DIDO workers were away, however the average amount of sleep when FIFO/DIDO workers were away was below the recommended 7 hours of sleep [44]. Given that the sample was mostly employed, with live-in dependents, managing multiple roles may be a contributing factor explaining this finding [45]. This is supported by participants’ answer to the open-ended question about “other factors that may affect your sleep” including: “*if I could nap I would but I have young children*”, “*stress of work and children*”, “*waking children*”, “*small children never allow sleep—its just life*”. Whilst FIFO/DIDO work arrangements were not found to be directly related to FIFO/DIDO partners’ sleep duration, extraneous factors such as increased household burden may be influencing factors and warrant further investigation. 

In comparison to the normative data of the Pittsburgh Sleep Quality Index (PSQI), findings indicated that FIFO/DIDO partners experienced poor sleep quality regardless of whether the FIFO/DIDO workers were at home or away [32]. Further, partners’ sleep quality was significantly poorer when FIFO/DIDO workers were away compared to when they were home. This finding is consistent with literature which showed that partners’ sleep quality was negatively influenced when FIFO workers were away [14]. In addition to sleep being a shared behaviour (i.e., influenced by the presence or absence of the FIFO/DIDO worker), it is also considered to be gendered, suggesting that women are more likely to be impacted by environmental and relationship stresses [45,46,47]. Current research has shown that FIFO/DIDO partners experience increased burdens of day-to-day decision making and difficulties transitioning from sole parenting to co-parenting across the FIFO/DIDO shifts [9,16]. 

The current study also found that loneliness may partially explain the difference in sleep quality across the FIFO/DIDO roster. Notably, the relationship between sleep quality and loneliness remained significant even after controlling for whether FIFO/DIDO workers were home or away, suggesting that loneliness impacts sleep quality beyond any consequential effects of the partner being home or away. Further, the finding that poorer sleep quality when workers were away compared to when they were home were not accounted for by factors such as having dependents in the home, relationship duration, chronotype, or duration in FIFO/DIDO role. These concurrent relational results provide some innovative insight into the potential impacts of FIFO/DIDO work arrangements on partners and families. However, further research is needed to understand the causality of the contributors and consequences of loneliness for the FIFO/DIDO mining population.

Co-sleeping data revealed that most participants almost always slept in the same bed as the FIFO/DIDO worker despite nearly all participants reporting some level of disruption to their sleep caused by their partner’s sleep habits (e.g., snoring, restlessness). This finding supports current literature suggesting that women often prioritise the sleep quality of their bed partner above their own sleep [45] and that sleep disturbances will be experienced by both bed partners [12]. Interestingly, however, participants were divided between getting better sleep when the FIFO/DIDO worker was away (38%) and getting better sleep when the FIFO/DIDO worker was at home (44%). This represents an interesting avenue for further research to explore how co-sleeping preferences impact sleep across the FIFO/DIDO roster. There was no clear indication of how long it took for participants’ sleep to return to normal after the FIFO/DIDO workers returned to work, however more participants reported sleep returned to normal after the first night (40%) when the FIFO/DIDO workers returned home. Thus, more research is needed to understand the transition periods for sleep immediately after FIFO/DIDO workers leave for work. 

FIFO/DIDO partners experience excessive daytime sleepiness compared to the normative range of the Epworth Sleepiness Scale (ESS) [22]. The majority of participants experienced mild to moderate excessive daytime sleepiness, whilst one third of participants reported severe sleepiness. There was only a slight decrease in participant sleepiness when FIFO/DIDO workers were away compared to when FIFO/DIDO workers were at home, although this difference was not significant. The creator of the ESS measure posits that ESS scores can be influenced by sleep disorders, ethnicity, and depression [48]. Current literature suggests that FIFO/DIDO partners experience greater depression when the FIFO/DIDO worker is away [15] and commentary provided in this study corroborated a prevalence of depression symptoms in this population, with a number of participants reporting depression (although this was not a direct measure nor was it accounted for in the present analyses). Nevertheless, the results demonstrate that FIFO/DIDO partners, in general, suffer from excessive daytime sleepiness compared to the general population. Results also offer further knowledge on the impacts of FIFO/DIDO work arrangements on partners and families, enabling an appreciation for the challenges experienced and informing strategies to improve the health and well-being of FIFO/DIDO partners.

When compared to normative data of the University of California, Los Angeles (UCLA) Loneliness Scale [28], most FIFO/DIDO partners experience moderate to extreme loneliness across the FIFO/DIDO roster. The findings support current literature where feelings of loneliness have been commonly reported by FIFO/DIDO partners [9,16,17]. The results indicate that FIFO/DIDO partners’ loneliness was greater when the worker was away compared to when the worker was at home. Sleep quality and sleep duration were found to be potential predictors of loneliness and may partially explain the difference in loneliness across the FIFO/DIDO roster. As described by others, loneliness can affect everyday life, decreasing sleep quality and increasing sleepiness independent of sleep duration [19]. The elevated levels of loneliness found in this study indicate that further research is needed to tease apart the impacts of loneliness on FIFO/DIDO partners’ health behaviours such as sleep. Research suggests that the relationship between loneliness and sleep is bidirectional, where increased loneliness perpetuates a vicious cycle of poor sleep quality, prolonged loneliness, and excessive daytime sleepiness [20]. The results found here suggest that FIFO/DIDO partners may be highly susceptible to this vicious cycle, with reported poor sleep quality, excessive daytime sleepiness, and increased feelings of loneliness compared to the general population, hence further research is needed to determine that causality of these variables and explore other potential contributors.

This study has several limitations, which should be acknowledged. First, subjective measures relying on self report may reflect response bias if the participant had difficulty understanding the question, separating home and away experiences, or reflecting on their relationship with their bed partner. The use of measures such as sleep diaries and objective sleep monitors may reduce the biases and should be implemented in future research. The recruitment of participants using FIFO/DIDO communities on social media was selected to achieve a large sample size in a relatively fast and inexpensive way. However, this online sampling method may have restricted participation from FIFO/DIDO partners who are not active in the FIFO/DIDO online communities. The convenience sample attained may be a limitation of this study and future research should consider a variety of recruitment methods to increase the representation of the target population. Of the 199 participants, 69 participants had started the survey and not completed it, resulting in high missing data rates. Rather than omit the data from participants who did not complete the survey, missing data was imputed using Fully Conditional Specification [37]. Although the missing data was imputed in a highly robust manner and the sample size was large for statistical power, it remains a limitation of this study. Whilst this study factored for age and sleep quality when examining sleep duration, the descriptive data and anecdotal evidence found further potentially influencing factors that warrant consideration. For example, how FIFO/DIDO partners manage multiple roles such as employment and raising young families should be considered in future research. The findings from the current study suggest that FIFO/DIDO partners may be completing a greater share of domestic responsibilities whilst maintaining employment responsibilities and this may be influencing sleep behaviours, as has been noted in other work arrangements such as on-call work [49,50,51]. Further research is needed on how their level of work and home responsibilities are associated with sleep behaviours and loneliness. In addition, anecdotal evidence indicated a presence of depression within the sample group. The ESS was used to measure sleepiness in participants. It should be noted that different forms of sleep disorders, ethnicity, and depression can affect the ESS scores [48]. These factors were not controlled for and are considered a limitation of this study herein. 

## 5. Conclusions

This research has found that FIFO/DIDO partners experience reduced sleep duration, poor sleep quality, elevated daytime sleepiness, and excessive levels of loneliness, with a significant difference in their sleep quality and loneliness when the FIFO/DIDO workers are away. No significant difference was found between when FIFO/DIDO workers were away and when FIFO/DIDO workers were at home for FIFO/DIDO partners’ sleep duration and sleepiness. The findings of this study provide a foundation on which future research can be conducted and not only within the mining population. These findings may also be applicable to other industries such as emergency services [43], offshore drilling [41], and transport [42], which require significant periods where a worker is away from home. Further exploration is necessary to understand the impacts of FIFO/DIDO work arrangements on FIFO/DIDO partners, although it is clear that sleep quality, sleep duration, sleepiness, and loneliness are at abnormal levels for the mining population as a whole. Further research should be conducted to examine the impacts of loneliness on sleep and performance and identify other potential influences so that appropriate coping strategies can be developed to improve FIFO/DIDO partners’ sleep habits and decrease feelings of loneliness in an effort to break the vicious cycle between sleep and loneliness. 

## Figures and Tables

**Table 1:** FIFO/DIDO Partners’ Sleep Quality, Sleep Duration, Sleepiness, and Loneliness.

	*n* Home	% Home	*n* Away	% Away
Sleep Quality (PSQI)				
Poor sleep (≥5)	165	82.9	185	93.0
Good sleep (<5)	34	17.1	14	7.0
Sleep Duration (PSQI)				
Less than 7 hours	64	32.3	85	42.9
7 hours or more	135	68.2	114	57.6
Sleepiness (ESS)				
Participant is getting enough sleep (<10)	17	8.5	21	10.6
Participant is suffering from excessive	111	55.8	106	53.3
daytime sleepiness (10–16)				
Participant is dangerously sleepy (>16)	71	35.7	72	36.2
Loneliness (UCLA)				
Extreme loneliness (≥30)	24	12.1	88	44.2
Moderate loneliness (22–29)	87	43.7	78	39.2
Normal loneliness (15–21)	62	31.2	28	14.1
Little to no loneliness (≤14)	26	13.1	5	2.5

Note. *n* = number of participants, % = percentage of participants, ESS = Epworth Sleepiness Scale, PSQI = Pittsburgh Sleep Quality Index, UCLA = University of California, Los Angeles.

**Table 2:** Summary of ANCOVA analyses comparing home versus away sleep quality, sleep duration, sleepiness, and lonliness.

Variable	M Home	SD Home	M Away	SD Away	*p* Home/Away
Sleep quality (PSQI)	7.8	3.3	8.8	3.1	0.001 *
Sleep duration (hrs)	7.2	1.5	6.9	1.6	0.486
Sleepiness (ESS)	15	4.1	15.4	4.6	0.960
Loneliness (UCLA)	22.2	6.2	27.9	6.4	<0.001 *

Note. * = statisically significant at *p* < 0.05. M = mean, SD = standard deviation, ESS = Epworth Sleepiness Scale, PSQI = Pittsburgh Sleep Quality Index, UCLA = University of California, Los Angeles.

**Table 3:** Results of Multiple Regression Analysis Predicting Sleep Quality.

	B	SEB	β	*t*	*p*
Intercept	9.58	1.37	--	7.01	<0.001*
Sleep Duration	−0.53	0.10	−0.25	−5.36	<0.001*
Home or Away	0.44	0.33	0.67	1.33	0.184
Dependents	−0.58	0.40	−0.07	−1.46	0.146
Relationship Duration	0.22	0.34	0.03	0.64	0.521
Chronotype	0.27	0.31	0.04	0.89	0.373
Duration in FIFO/DIDO Role	−0.41	0.20	−0.10	−2.03	0.043 *
Loneliness	0.12	0.03	0.25	4.83	<0.001 *

Note. B = unstandardized regression coefficient; SEB = Standardised error of the coefficient; β = standardised coefficient; * = significant at *p* < 0.05.

## Abbreviations

ESS	Epworth Sleepiness Scale
FIFO/DIDO	Fly in Fly out, Drive in Drive Out
PSQI	Pittsburgh Sleep Quality Index
UCLA	University of California, Los Angeles
